# Morphological and Genetic Diversities of *Habenaria radiata* (Orchidaceae) in the Kinki Area, Japan

**DOI:** 10.3390/ijms22010311

**Published:** 2020-12-30

**Authors:** Tsutomu Tachibana, Yuki Nishikawa, Nakao Kubo, Seiji Takeda

**Affiliations:** 1 Graduate School of Life and Environmental Sciences, Kyoto Prefectural University, Kyoto 606-8522, Japan; t.tachibana0729.aquaterrarium@gmail.com (T.T.); yuuki100smile@gmail.com (Y.N.); nkubo@kpu.ac.jp (N.K.); 2 Biotechnology Research Department, Kyoto Prefectural Agriculture, Forestry and Fisheries Technology Center, Seika, Kyoto 619-0244, Japan

**Keywords:** *Habenaria radiata*, hawkmoth, lip (labellum), microsatellite, Orchidaceae, phylogeny

## Abstract

Floral organs have evolved from leaves for reproduction, and the morphological analyses help to understand the plant diversity and evolution. *Habenaria radiata* (syn. *Pecteilis radiata*) is a terrestrial orchid living in wetlands in Japan, Russia, South Korea, and China. The habitats of this plant in Japan have been reduced because of environmental destruction and overexploitation, and thus it is on the Red List of Japan as a Near Threatened species. One of the three petals of the *H. radiata* flower is called a lip or labellum, which resembles a flying white bird, egret, or white heron, with its proposed function being to attract pollinators. To understand the diversity of *H. radiata* plants in different areas, we examined the lip morphology and phylogeny of populations from eight habitats in the Kinki area, Japan. The complex shapes of the lips were quantified and presented as a radar chart, enabling characterization of the morphological difference among populations. Phylogenetic analysis with microsatellite markers that we generated showed the variation of genetic diversity among populations, suggesting the different degrees of inbreeding, outbreeding, and vegetative propagation. Our approach offers a basic method to characterize the morphological and genetic diversity in natural populations.

## 1. Introduction

Floral organs have evolved from leaves to have various shapes that facilitate reproduction. The petals in many plants develop to have unique shapes to attract pollinators. The flower shapes in orchids especially vary widely among species, and have established close relations with pollinators; thus, the petal morphology in orchids is critically important for the reproduction.

Orchidaceae is one of the largest families of angiosperms, containing more than 28,000 species [1]. *Habenaria* is a large genus of orchids with more than 850 species living in the tropical and subtropical regions [2,3]. *Habenaria radiata* (Thunb.) Spreng., Syn. *Pecteilis radiata* (Thunb.) Raf. is a terrestrial orchid living in the wetlands in Honshu, Kyushu, and Shikoku in Japan, the Far East of Russia, South Korea, and China (World Checklist of Selected Plant Families, https://wcsp.science.kew.org/, accessed on 1 December 2020). In recent years, the habitats of *H. radiata* have been reduced because of environmental destruction and overexploitation, and thus this species is registered as a Near Threatened species in the Red List in Japan (Ministry of the Environment, Red List 2020, https://ikilog.biodic.go.jp/Rdb/booklist, accessed on 1 December 2020).

**Figure 1:**
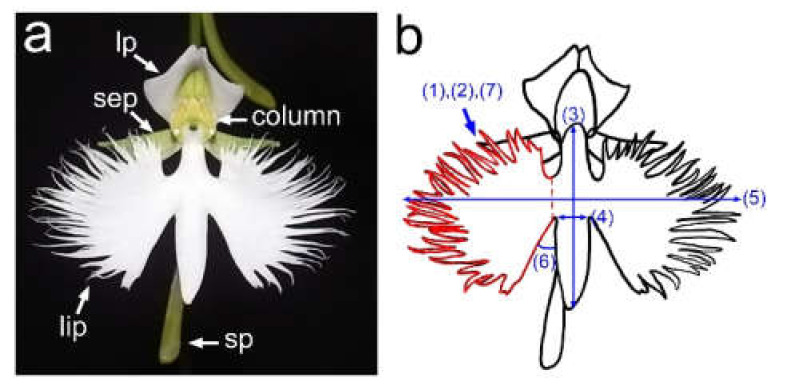
(**a**) *H. radiata* flower. Note: lip, lip (or labellum); lp, lateral petal; sep, sepal; sp, spur. (**b**) Seven measurement factors used to quantify the lip shape: (1) area, (2) perimeter, (3) body length, (4) body width, (5) wing length, (6) angle between body and wing, and (7) serration number.

*H. radiata* flowers consist of three sepals, two lateral petals, one lip (or labellum), and one column (Figure 1a). A spur, a long tubular structure, is formed at the base of the lip and accumulates nectar in the apical end. The lip consists of a body-like middle part and two wing-like lobes; this shape resembles a flying egret (white heron). The Japanese name for this plant “Sagi-sou” (meaning an egret plant), which is derived from the lip morphology, and it has been described in many literature works and novels in Japan. The complex lip shape may play a role in attracting pollinators such as skippers and hawkmoths [4,5,6]. It is suggested that *H. radiata* carries local variation in flower shape in different habitats [7], however quantitative and genetic analyses of the variation have not been performed before.

Molecular markers are powerful tools used to investigate the genetic variation in populations. Microsatellites or simple sequence repeat (SSR) markers are relatively easy to develop and useful for characterizing genetic differences based on genomic DNA sequences. An SSR marker is a short tandem repeat consisting of 1 to 6 nucleotides in the genome, whose repeat numbers can differ among individuals. Molecular markers raised by the SSRs have been widely used for genetic breeding, genome mapping, population genetics, and taxonomic and phylogenetic studies [8,9]. In Orchidaceae, SSR markers have been used to analyze genetic diversity [10,11,12], and among the *Habenaria* genus, the genetic diversity of *H. edgeworthii*, which was used as an Indian medicine, has been examined with inter-simple sequence repeat (ISSR) markers [13].

To understand the diversity of *H. radiata*, we examined the morphology and phylogeny of populations from eight habitats in the Kinki area, Japan. Quantification of lip shape by eight indices showed the differences in flower morphology among the populations. We developed novel SSR markers and analyzed phylogenetic relationships, and showed the genetic diversity of *H. radiata* populations. Our results offer basic methods to characterize the morphological and genetic diversity in natural populations.

## 2. Results

### 2.1. Quantification of Lip Shape

**Figure 2:**
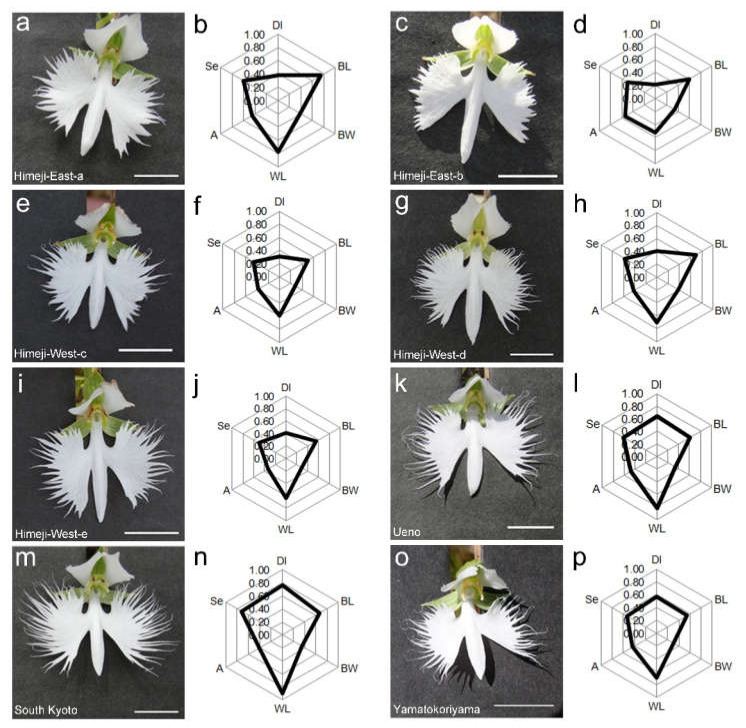
Flowers and quantification of lip shapes of *H. radiata* from eight habitats in the Kinki area. (**a**,**c**,**e**,**g**,**i**,**k**,**m**,**o**) Flowers of each population. Scale bars: 1 cm. (**b**,**d**,**f**,**h**,**j**,**l**,**n**,**p**) Quantification of lip shape shown by six parameters. DI, dissection index; BL, body length; BW; body width; WL, wing length; A, angle; Se, serration number. (**a**,**b**) Himeji East–a (*n* = 6). (**c**,**d**) Himeji East–b (*n* = 8). (**e**,**f**) Himeji West–c (*n* = 3). (**g**,**h**) Himeji West–d (*n* = 17). (**i**,**j**) Himeji West–e (*n* = 6). (**k**,**l**) Ueno (*n* = 5). (**m**,**n**) South Kyoto (*n* = 14). (**o**,**p**) Yamatokoriyama (*n* = 10).

To understand the diversity among different populations, *H. radiata* plants were collected from eight populations in the Kinki area. During collection, we noticed that the morphologies of the flowers, especially the lips, showed variation among populations. Since the lip shapes are complex, we quantified them by measuring several values and showed these with radar charts, as well as graphs showing each data point (Figure 1b, Figure 2 and Figure S1; see Section 4). Statistical analysis showed that the South Kyoto population had the largest and most complex lips, as shown by area, perimeter, and dissection index. Himeji populations had shallower serration compared to the other regions; Himeji–East–b lips especially were the smallest and had the least complex periphery. We propose that this quantification of lip shape is a useful tool to characterize the morphologies of local populations of *H. radiata*.

### 2.2. Phylogenetic and Genetic Analyses

To elucidate the genetic variation among populations, we developed microsatellite (SSR) markers. The SSR-enriched libraries from *H. radiata* genomic DNA were constructed. The 144 clones were screened from the libraries for sequencing, resulting in 107 SSR-containing clones (74.3%). Based on this, we designed 82 SSR primers, and after elimination of unsuccessful primers, 19 SSR primers detecting 21 loci were selected (Table S1). Five markers showed significant differences from the Hardy–Weinberg equilibrium (HWE), probably due to the small number of examined plants. In examined populations, the expected (*He*) and observed (*Ho*) heterozygosities were in the ranges of 0.2362 to 0.6410 and 0.3187 to 0.6139, respectively (Table 1 and Table S2). The fixation index (*F*) values in Ueno were significantly higher than the HWE expectation, suggesting a high frequency of inbreeding within the population. On the other hand, those in Himeji East–a, South Kyoto, and Yamatokoriyama were significantly low, suggesting excess heterozygosity in these populations (Table 1). Since *H. radiata* propagates both with seeds and bulbs, excess heterozygosity may be due to vegetative propagation after a previous bottleneck or to a high frequency of outbreeding by pollinators within the restricted area.

**Figure 3:**
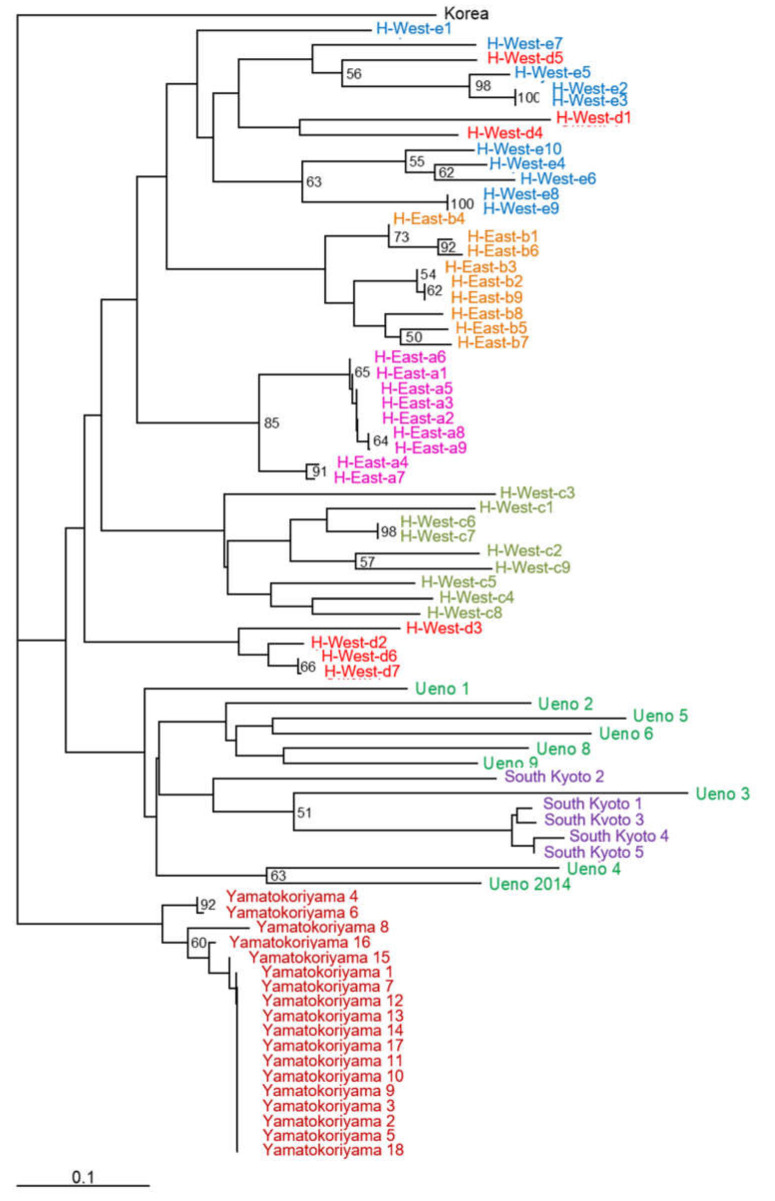
Neighbor-joining phylogenetic tree of *H. radiata* collected from the Kinki area. Numbers at the nodes are bootstrap values from 1000 replications (>50%). Scale bar: genetic distance [14].

Phylogenetic analysis showed that *H. radiata* populations were classified into three groups: Yamatokoriyama, Himeji, and Ueno and South Kyoto (Figure 3). Himeji West–d plants were separated into two subgroups of the other Himeji west area, suggesting outcrossing between these habitats. Plants from Ueno and South Kyoto were in the same group, suggesting that they share common ancestors.

**Figure 4:**
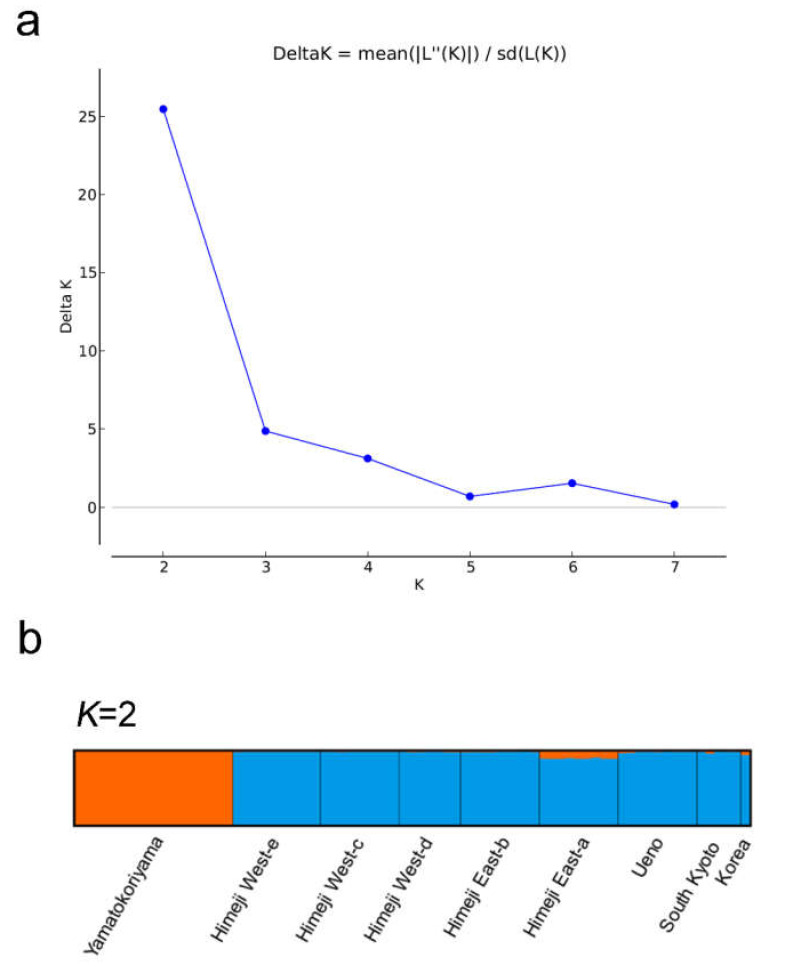
Hierarchical genetic population structure analysis of *H. radiata*. (**a**) Estimation of the optimal *K* value. (**b**) Result at the optimal value (*K* = 2). Populations are separated by vertical lines, whose names are listed below bar plots. Orange and blue colors of bar plots correspond to the sum of assignment probabilities from the clusters at each subgroup.

The STRUCTURE analysis showed that the optimal number of cluster (*K*) values was 2 and the populations were classified into two genetic pools (Figure 4). The phylogenetic and STRUCTURE analyses suggested that the Yamatokoriyama population was genetically separated from the other populations.

### 2.3. Hawkmoth Hovering during Sucking of Nectar from H. Radiata Flowers

The complex shape of the lips suggests that the lips of *H. radiata* act as visible markers or landing places. It is known that two skipper species (*Pelopidas mathias* and *P. guttata*) and one hawkmoth (*Theretra japonica*) visit *H. radiata* flowers and transfer pollinia by attaching them on their head [4,5,6]. We recorded flower visitors with one video camera and found that the hawkmoth (*Theretra oldenlandiae*) visited several flowers and seemed to suck nectar from spurs while hovering, without landing on the flowers (Movie S1). This suggests that the lip acts as a visible marker for flying hawkmoths. It is possible that they hold the lips with their limbs during sucking, but we were not able to confirm this from our recordings.

## 3. Discussion

We have shown methods for the quantification of complex lip shapes and phylogenetic analysis of *H. radiata*, an endangered wild orchid in Japan. These methods allowed us to investigate intra- and inter-population variations of *H. radiata*, and therefore are useful for characterizing a population.

One of the most remarkable features in *H. radiata* flowers is the lip morphology. Most orchids are believed to have evolved their flower morphology to attract pollinators. Some species develop a spur and accumulate nectar as a reward for visitors, while the others give no rewards and attract visitors by deception with scent; color; or shapes that mimic the female insect morphology, food, or nest [15]. *H. radiata* flowers develop a long tubular spur and accumulate nectar within it, and thus are a rewarded-type flower. We suggest that the lips function as visible markers for hawkmoths. Quantification of the lip morphology suggests that the dissection index (DI) values, representing the complexity of the lip shape, are different between the Himeji region and the others. The former’s flowers have a more simple shape (DI: ~0.04), whereas the latter display values of 0.6~0.8 (Figure 2). The morphologies of the lips varied among populations (Figure 2), suggesting variation of pollinators along with that of lips in different habitats, e.g., variations in body size and preference for lip shape. Investigation of pollinator variations in different habitats is required to confirm this hypothesis.

Phylogenetic analysis by microsatellite markers showed the relation among populations. Himeji populations were classified into one subfamily, although within this subfamily the populations were not divided into the east and west groups. The east and west areas in Himeji city are more than 10 km away from each and are separated by a big city. Within the east or west area, each habitat was about 3 km away from the others. The West–d plants were subdivided into two groups in the west area, suggesting a possibility of outbreeding within these populations, which may be achieved by flying pollinators such as hawkmoth and skippers. It is unusual that plants in East–b, West–e, and some from West–d were classified into the same subfamily. Additionally, Ueno and South Kyoto plants were in the same subfamily, although they are about 10 km away. It is possible that natural pollinators can travel for long distances between these areas, or the plants were transplanted by humans, since sometimes people transplant endangered plants in nature for conservation. Yamatokoriyama plants was genetically separated from the other populations and showed significantly lower *F* values than the HWE expectation. This habitat has been taken care of for several years, and it was confirmed that the plants were once reduced dramatically due to damage by a typhoon. This suggests that this population went through a bottleneck and has been propagated by bulbs, keeping a high ratio of heterozygosity.

*H. radiata* is a wetland plant and is designated as an endangered species in 43 prefectures in Japan (http://jpnrdb.com/, accessed on 1 December 2020). This may be because of the reduction of wetland habitats due to excessive land development and overexploitation. Our methods can be used to characterize the population, and thus may help to maintain the local population in natural habitats. This work was limited to the populations in the Kinki area, and we need to examine the other populations left in nature. We also need to think about other natural species living in wetlands to conserve the natural conditions for our future.

## 4. Materials and Methods

### 4.1. H. radiata Populations and Growth Conditions

*H. radiata* plants were sampled from eight habitats with permission: five from Himeji (two and three from the west and east areas, respectively), and the others from Yamatokoriyama (Nara), South Kyoto, and Ueno Forest Park (Mie). The authors apologize that the details of the habitats are withheld to avoid overexploitation. The information is available upon request, although we may not be able to respond depending on the request content. Note that gathering of any plants and animals is prohibited in these area and parks without permission. For the recording of flower visitors, bulbs were sown on cultivation soil (Nippi Engeibaido 1 go, Nihon Hiryo Co. Ltd., Fujioka, Japan) covered with wet sphagnum and grown outside at the Seika campus, Kyoto Prefectural University, Seika, Kyoto.

### 4.2. Quantification of Lip Morphology

To quantify the complex shapes of the lips, we defined the middle part of a lip as the body and the two lobes as wings, then measured the dissection index (perimeter/√area) values of the left wing, body length, body width, wing length, angle between body and left wing, and serration number of the left wing (Figure 1b and Figure S1). The ratio of these values was divided by each max value (dissection index: 40; body length: 30; body width: 10; wing length: 40; angle: 90; serration number: 50), shown as radar charts in Figure 2.

### 4.3. DNA Extraction, Generation of SSR Markers, and PCR Amplification

Genomic DNA samples were extracted from young leaves at approximately 100 mg using a DNeasy Plant Mini Kit (QIAGEN, Hilden, Germany). *H. radiata* genomic DNA for the Korean population [16] was used as the outgroup sample. SSR-enriched libraries were constructed from *H. radiata* genomic DNA as reported previously [17]. PCR primers were designed from the flanking sequence of the SSR sequence. The 5’-end of the forward primer was labeled with D2–D4 fluorescent dye (Sigma–Aldrich, St. Louis, MO, USA and Alpha DNA, Montreal, Canada). PCR amplifications were performed using a KAPA Taq Extra PCR Kit (Kapa Biosystems, Wilmington, DE, USA) with the conditions of initial denaturation at 94 °C for 5 min, 30 cycles of 94 °C for 30 s, 50 or 52 °C (Table S1) for 1 min, and 72 °C for 1 min, followed by final extension at 72 °C for 5 min. The DDBJ/EMBL/GenBank database accession numbers of the nucleotide sequences used for SSR primer design are LC316947–LC346964 (Table S1).

### 4.4. Phylogeny and Genetic Diversity

The genetic parameters of the expected heterozygosities (*He*), observed heterozygosities (*Ho*), and fixation index (*F*) values were calculated using Genepop version 4.2 [18,19]. Hardy–Weinberg equilibrium and null allele frequency values were calculated with CERVUS 3.0 [20]. Fragment sizes were analyzed with a CEQ8000XL DNA sequencer (Beckman Coulter, Brea, CA, USA), and treated as allele data. A neighbor-joining (NJ) phylogenetic tree was constructed based on Nei’s standard genetic distance [14] using POPULATIONS software version 1.2.32 [21]. Bootstrap analysis was performed from 1000 replications. The tree was visualized using TREEVIEW [22]. The population structure was analyzed using the STRUCTURE software [23]. The numbers of clusters (*K*) were set from 1 to 8 and each *K* was run 10 times. Each run was carried out with a burn-in period of 50,000 and 1,000,000 MCMC replications. The online STRUCTURE HARVESTER software was used to estimate optimal *K* values [24]. These results were visualized using the online software CLUMPAK [25].

### 4.5. Pollinator Recordings and Imaging Analysis

Pollinators were recorded with a JVC video camera (Everio R, JVC, Yokohama, Japan) in the morning or evening on 18 days from 9 July to 3 August 2018. The total recording time was 85 h, 56 min, 35 s. Flowers and petals were captured with a digital camera (GRII, RICHO, Tokyo, Japan) and quantified with ImageJ software [26].

## Figures and Tables

**Table 1:** Heterozygosity of each population.

Population	*N*	*H_e_*	*H_o_*	*F*
Himeji East-a	9	0.2583	0.4409	−0.7067 *
Himeji East-b	9	0.2695	0.3187	−0.1825
Himeji West-c	9	0.4103	0.3533	0.1390
Himeji West-d	7	0.4445	0.5180	−0.1654
Himeji West-e	10	0.4380	0.4203	0.0404
Ueno	9	0.6410	0.5318	0.1731 *
South Kyoto	5	0.4311	0.6139	−0.4239 *
Yamatokoriyama	18	0.2362	0.4441	−0.8803 *

*N*, number of individuals; *He*, expected heterozygosity; *Ho*, observed heterozygosity; *F*, fixation index; asterisks, significant deviations from the Hardy–Weinberg equilibrium expectations (*p* < 0.05).

## Data Availability

The data presented in this study are available on request from the corresponding author.

## Supplementary Materials

Supplementary materials can be found at https://www.mdpi.com/1422-0067/22/1/311/s1.

## Abbreviations

MDPI	Multidisciplinary Digital Publishing Institute
DOAJ	Directory of open access journals
TLA	Three letter acronym
LD	Linear dichroism
