# Supplementary material for: Morphological and Genetic Diversities of Habenaria radiata (Orchidaceae) in the Kinki Area, Japan
Source: Int J Mol Sci. 2020 Dec 30;22(1):311. doi: 10.3390/ijms22010311 (PMC7795838; doi:10.3390/ijms22010311)
Supplement: Supplementary file 1 [file ijms-22-00311-s001.zip › Supplementary files/Supplementary Figs_final.docx]

**
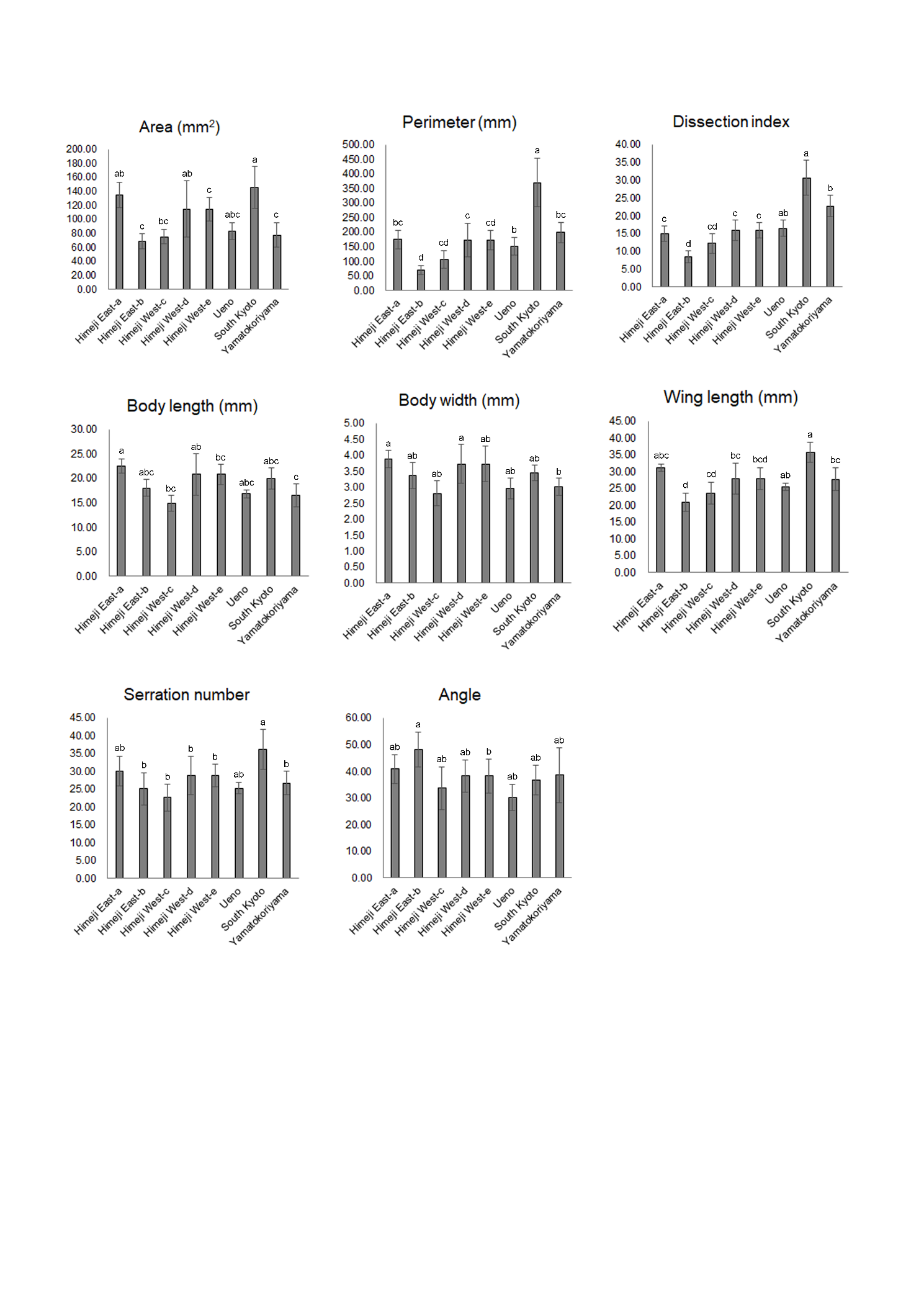
**

**Figure S1**. Quantification of lip morphology shown by each parameter. Data represent means ± SD and graph bars with the same letters are not significantly different (P<0.05) analyzed by Tukey–Kramer multiple comparison test.

**Movie S1**. A movie capturing a hawkmoth visiting flowers of *H. radiata*. Note that the movie is dark because it was recorded in the late evening, so authors ask readers to gain the brightness and contrast with the media player application, e.g. VLC media player.
